# Green tea extract attenuates LPS-induced retinal inflammation in rats

**DOI:** 10.1038/s41598-017-18888-5

**Published:** 2018-01-11

**Authors:** Jia Lin Ren, Qiu Xiao Yu, Wei Cheng Liang, Pui Ying Leung, Tsz Kin Ng, Wai Kit Chu, Chi Pui Pang, Sun On Chan

**Affiliations:** 1 School of Biomedical Sciences, Faculty of Medicine, The Chinese University of Hong Kong, Shatin, N.T. Hong Kong SAR, China; 2 Department of Ophthalmology and Visual Sciences, Faculty of Medicine, The Chinese University of Hong Kong, Kowloon, Hong Kong SAR China

## Abstract

Inflammation is in a wide spectrum of retinal diseases, causing irreversible blindness and visual impairment. We have previously demonstrated that Green Tea Extract (GTE) is a potent anti-inflammatory agent for anterior uveitis. Here we investigated the anti-inflammatory effect of GTE on lipopolysaccharides (LPS)-induced retinal inflammation in rats and explored the underlying mechanism. Adult rats were injected with LPS and GTE was administered intra-gastrically at 2, 8, 26 and 32 hours post-injection. Staining of whole-mount retina showed that the number of activated microglia cells was significantly increased at 48 hours post-injection, which was suppressed after GTE treatment in a dose-dependent manner. Activation of astrocytes and Müller glia in the retina was also suppressed after GTE treatment. Meanwhile, GTE reduced the expression of pro-inflammatory cytokines including IL-1β, TNF-α and IL-6 in retina and vitreous humor. These anti-inflammatory effects were associated with a reduced phosphorylation of STAT3 and NF-κB in the retina. Furthermore, the surface receptor of EGCG, 67LR, was localized on the neurons and glia in the retina. These findings demonstrate that GTE is an effective agent in suppressing LPS-induced retinal inflammation, probably through its potent anti-oxidative property and a receptor-mediated action on transcription factors that regulate production of pro-inflammatory cytokines.

## Introduction

Tea is an agricultural product and the second most popular drink in the world. Green Tea Extract (GTE) consists of catechins as the major polyphenols, which include (−)-epicatechin (EC), (−)-epigallocatechin (EGC), (−)-epicatechin gallate (ECG), and (−)-epigallocatechin-3-gallate (EGCG)^1–3^. Among them, EGCG is the most abundant constituent, accounting for over 50% of the total catechins^4^. EGCG has been shown to exert the anti-cancer and anti-inflammatory functions through its cell surface receptor 67 Laminin receptor (67LR)^5–7^. Previous studies of GTE have indicated a preventative effect of green tea in relieving the severity of obesity, neurodegeneration, and cardiovascular diseases^8–12^. We previously demonstrated that GTE is a potent anti-inflammatory and anti-oxidative agent in the eye, alleviating the endotoxin-induced acute anterior uveitis and sodium iodate-induced retinal degeneration in rats^13,14^, suggesting a potential treatment for ocular diseases.

Retinal inflammation can be associated with visual impairment and even total vision loss. It is a common pathology in different retinal diseases, including uveitis, age-related macular degeneration (AMD) and diabetic retinopathy^15^. Currently, administration of corticosteroid is the standard therapeutic strategy but long-term use carries potential risks of formation of cataract and development of glaucoma. Hence, alternative treatments with less severe side effects must be sought^16^.

Endotoxin-induced uveitis (EIU) is a classic animal model to study human acute anterior uveitis^17^. The ocular inflammation can be induced by systemic injection of lipopolysaccharide (LPS), an endotoxin isolated from Gram-negative bacteria. We have shown that acute inflammatory responses develop in 24 hrs in iris and ciliary body following induction with LPS, while a mild inflammation is also observed in posterior segments of the eye, including the retina^17,18^. Oral administration of GTE reduces significantly infiltration of inflammatory cells and exudation of proteins in the aqueous humor, which are associated with reduced expression and secretion of proinflammatory factors in the ciliary body and iris^13^. In the present study, we showed that LPS could be used to establish a retinal inflammatory model on SD rats as well. Hence, the aim of current study was to provide an overview of the retinal inflammation induced by LPS and suggest a potential beverage to minimize the consequences of the retinal inflammation.

## Results

### GTE reduces LPS-induced infiltrating cells in posterior segment of the eye

**Figure 1:**
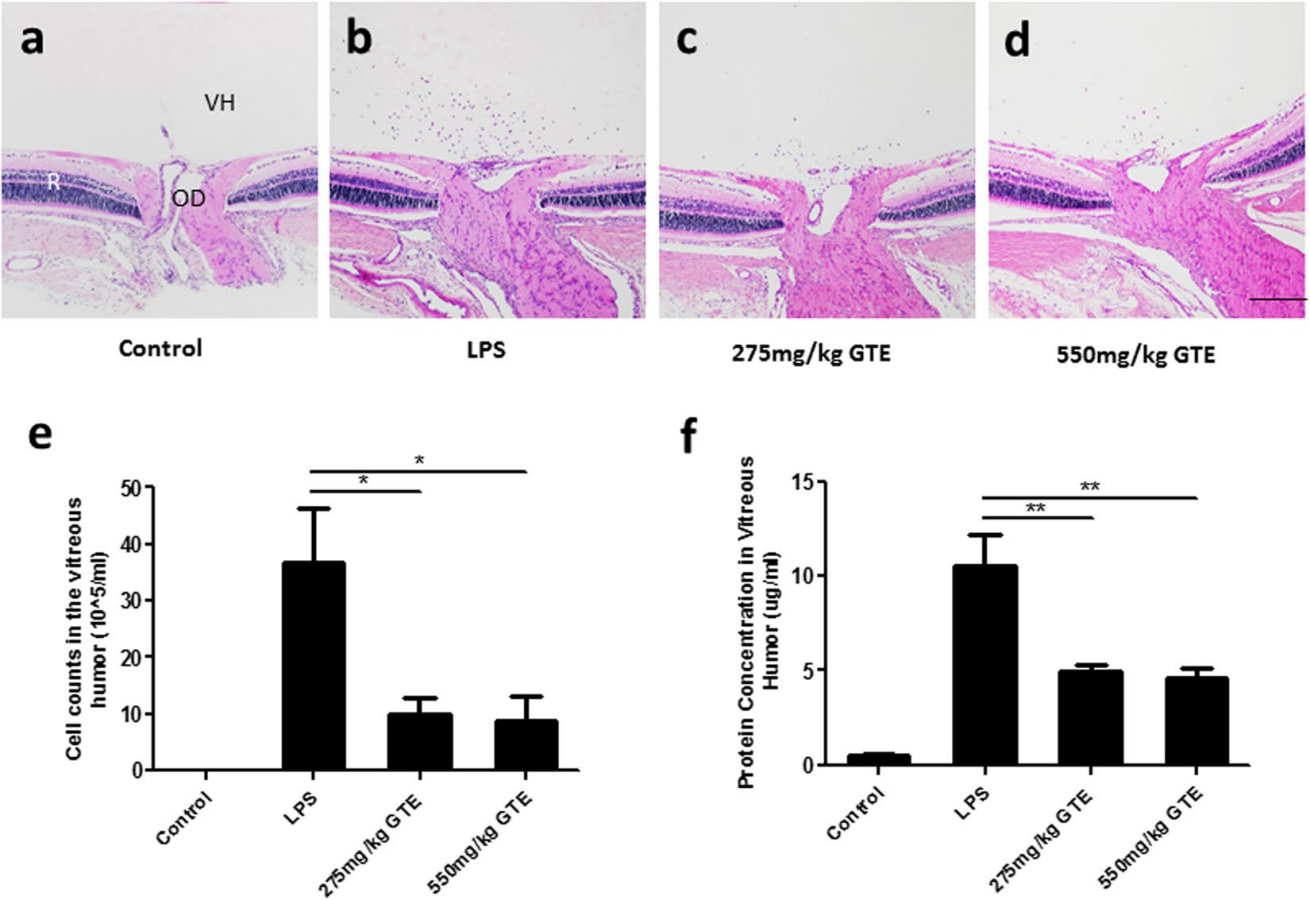
Green tea extract suppressed the infiltrated cell number and protein accumulation in vitreous humor. (**a**,**b**) H&E sections showed that a large amount of infiltrated cells aggregated around the optic disc (OD) at 48 hrs after LPS stimuli. (**c**,**d**) Both 275 mg/kg GTE and 550 mg/ml GTE supplement after LPS stimulation decreased the cell number accumulated in vitreous humor (VH) dramatically. Scale bar 200 µm. (**e**) Analysis of vitreous humor demonstrated that the number of infiltrated cells and protein concentration was reduced dramatically in LPS injected rats after GTE administration. *p < 0.05, **p < 0.01, n = 7 in each group.

Ocular inflammation was induced in adult rats by injecting 1 mg/kg LPS into the left footpad. Histological section of the eye showed that LPS induced substantial accumulation of infiltrating cells in the vitreous humor 48 hrs after injection (n = 7) (Fig. 1b), which was not detected in the saline-injected rats (n = 7, Fig. 1a). The acute inflammation did not cause any detectable morphological changes in the retina at 48 hours post-injection by the histological sections. GTE treatments (oral gavage, 4 times at 2, 8, 26, and 32 hours after LPS injection) at the dose of 275 mg/kg produced an obvious reduction of inflammatory cells in the vitreous (Fig. 1c) of LPS injected rats and this reduction was more obvious in rats that had received double dose of GTE (550 mg/kg) (Fig. 1d). We also analyzed the infiltrated cell number and protein concentration of vitreous humor extracted from the eye. The result showed that the number of inflammatory cells were reduced significantly after GTE treatments, both at the dose of 275 and 550 mg/kg (9.77 ± 3.17 and 8.75 ± 4.48 × 10^5^ cells/ml, respectively), when compared with that collected from rats with LPS injection alone (39.73 ± 9.74 × 10^5^ cells/ml, p < 0.05) (Fig. 1e). Meanwhile, the protein accumulated in vitreous humor was suppressed by 275 and 550 mg/kg GTE administration (4.96 ± 0.33 and 4.61 ± 0.47 μg/ml, respectively) compared with LPS group (10.50 ± 1.65, p < 0.01 μg/ml) (Fig. 1f).

### GTE ameliorates the activation of microglia and Müller glia in the retina

**Figure 2:**
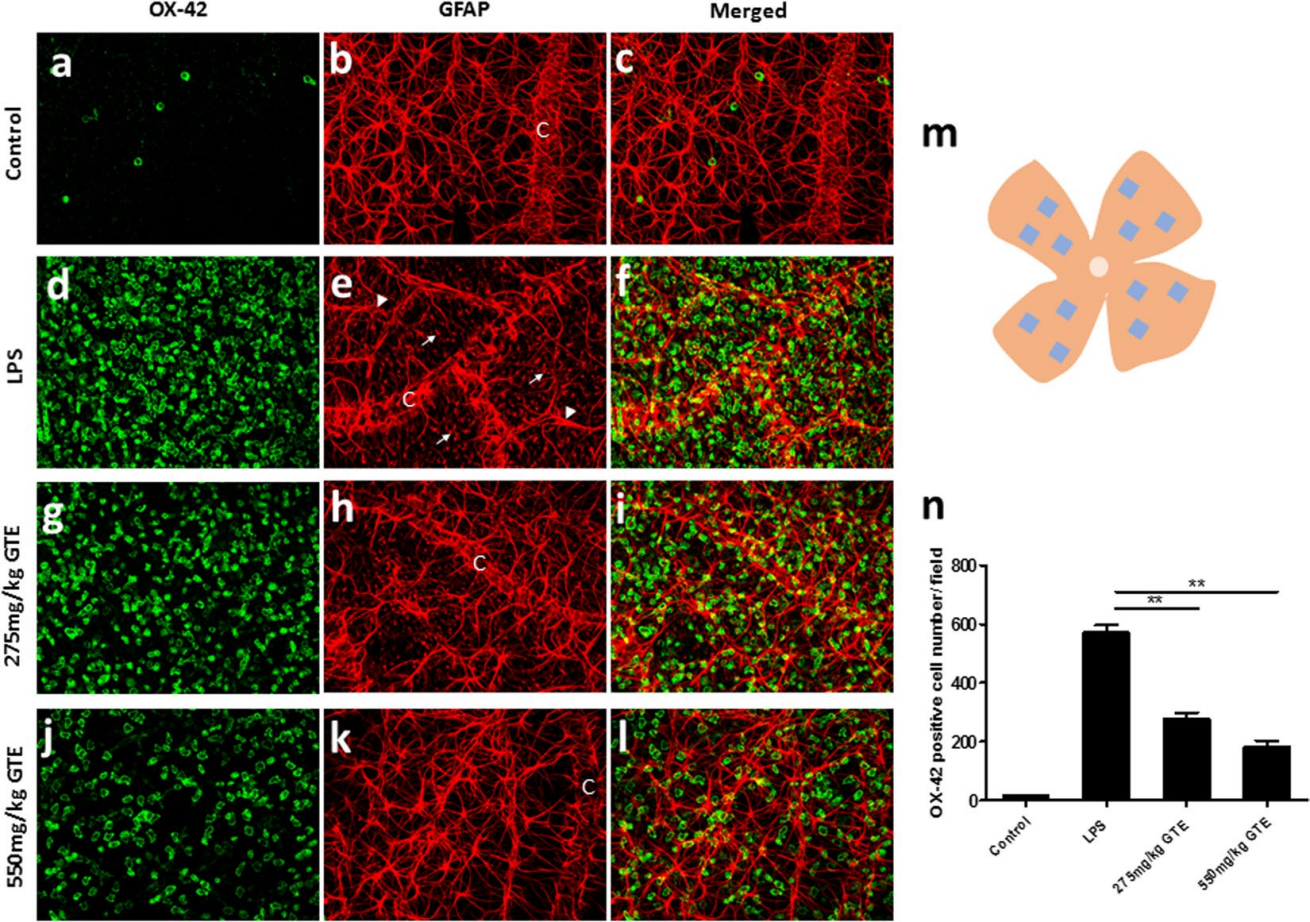
Green tea extract reduced the number of activated microglia in ganglion cell layer. (**a**–**c**) Only a few OX-42 positive microglia cells and well-organized astrocytes network were observed in control retina. (**d**–**f**) After LPS treated, numerous microglia cells emerged in the retinal ganglion cell layer and the processes of astrocytes were disrupted. Meanwhile, some astrocytes became hypertrophied (arrowhead) and the endfeet of Müller glia cells (white arrow) appeared as well. Moreover, the glial endfeet along the capillary (C) were highly disorganized. GTE treatment at the dosage of 275 mg/kg (**g**–**i**) and 550 mg/kg (**j**–**l**) reduced the number of microglia cells and improved the disordered condition of astrocytes. The astrocytic array and blood-retinal barrier was preserved, especially under high dose group (**k**). Scale bar, 40 µm. (**m**) A total of 12 areas were imaged from each retina flat mount to calculate an average number of the immunoreactive microglia cells. (**n**) The number of activated microglia cells was significantly suppressed by the treatment of GTE in both 275 mg/kg and 550 mg/kg dosage. **p < 0.01, n = 6.

Astrocytes, microglia and Müller glia cells are three predominant types of glia cell in retina. Astrocytes are localized in GCL and their endfeet play a vital role in the maintenance of blood–retina barrier^19^. Microglia are the resident macrophage cells in central nervous system and extremely sensitive to various pathological changes^20^. Both of them will undergo morphological changes in inflammation and produce some pro-inflammatory cytokines such as TNF-α and IL-1β^21^. The response of astrocytes and microglia were examined in the retina during this acute inflammation. The retina wholemounts were stained simultaneously with OX-42 antibody for microglia and anti-GFAP for astrocytes 48 hrs after LPS injection. In the control retina, only a few microglia cells (14.3 ± 4.26 cells per field) were observed in a field of the ganglion cell layer of the wholemount, where it was populated densely with astrocytes that form a highly organized array (Fig. 2a–c). Astrocytes adjacent to the blood vessels extended end-feet to the capillaries, contributing to the blood-retinal barrier (Fig. 2b). Injection with LPS caused a dramatic increase of microglia (571.47 ± 25.55 cells per field) in the retinal ganglion cell layer (GCL) (Fig. 2d) and the astrocytic array was disrupted (Fig. 2e), with astrocyte hypertrophy and appearance of Müller glial processes. Moreover, the glial endfeet along the capillary was highly disorganized, suggesting a disruption of the blood-retinal barrier during this acute inflammatory process (Fig. 2e,f). Treatments of GTE at the dose of 275 mg/kg (Fig. 2g–i) or 550 mg/kg (Fig. 2j–l) produced obvious reduction of microglial cells (275.93 ± 21.45 and 180.82 ± 22.42 cells per field, respectively) and suppression of astroglial reactions. The astrocytic array and the glial endfeet on the blood vessels were restored, particularly under high dose of GTE (Fig. 2k). The OX-42 immunoreactive microglial cells were counted in these retinal wholemounts (Fig. 2m). Analysis of the cell number demonstrated a significant reduction of microglial cells after GTE treatments when compared with rats injected with LPS (P < 0.01), and that this reduction was dose dependent (Fig. 2n).

**Figure 3:**
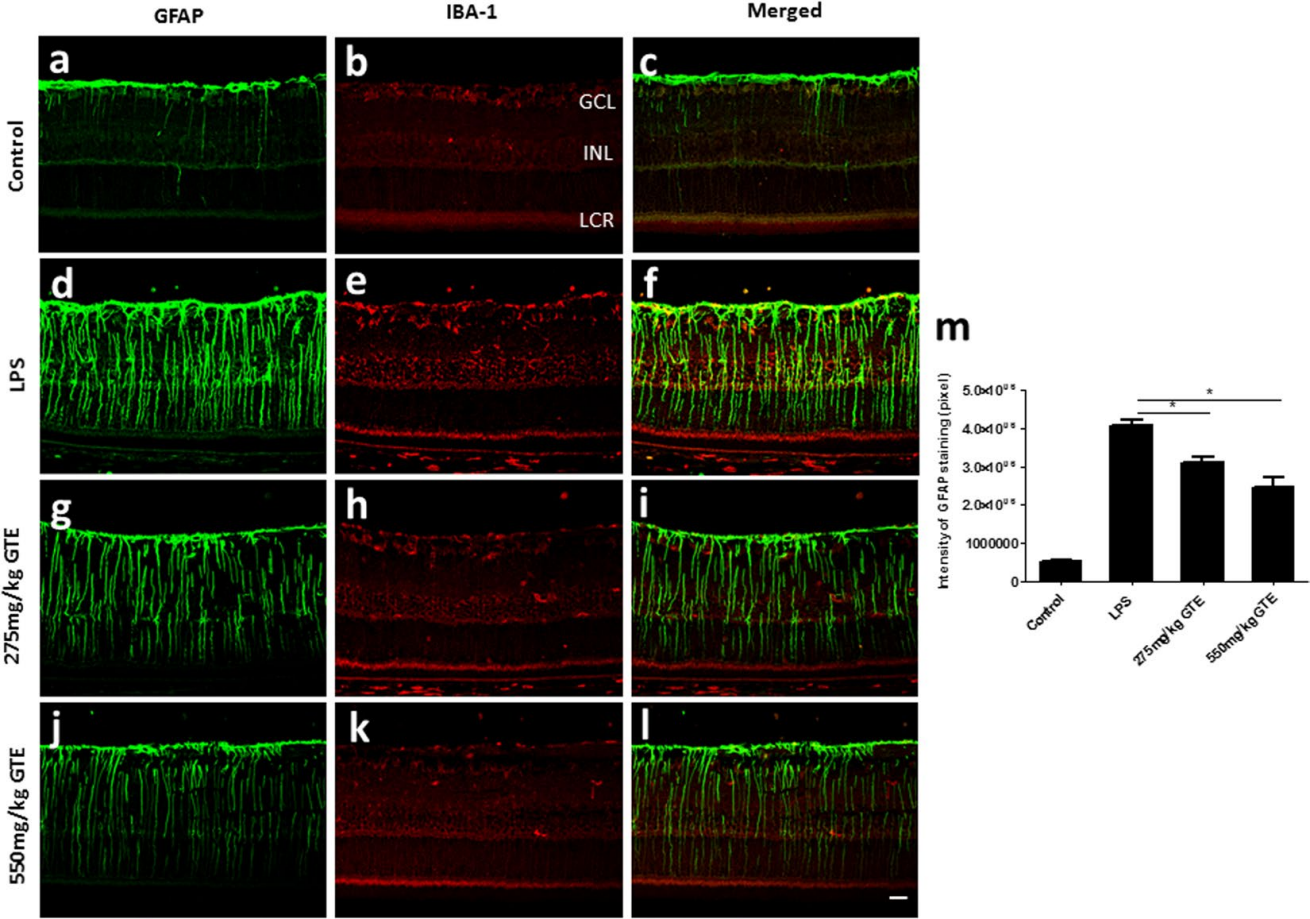
Green tea extract attenuated the activation of Müller glial cells. (**a**–**c**) GFAP staining labelled a dense layer of astrocytes and a few Müller glia processes that run through the whole thickness of the retina in control group. (**b**) IBA-1 staining showed the localization of a few microglia in the ganglion cell layer (GCL) and inner nuclear layer (INL). (**c**–**f**) After LPS insult, the expression of GFAP in Müller glia cells was elevated obviously, which accompanied by an increase in microglial cells in GCL and INL. (**g**–**l**) GTE treatments attenuated the intensity of GFAP staining and density of IBA-1 staining dramatically, especially in high dose group. (**m**) Measurement of the fluorescence intensity in these tissues showed GTE produced a dose-dependent reduction of GFAP immunoreactivity. *p < 0.05, n = 6.

The glial reactions after LPS treatment were also investigated in cross-sections of the retina. In the control retina, a dense layer of GFAP-positive astrocytes was observed in the RGC layer with a few Müller glial processes extending to inner nuclear layer (INL) (Fig. 3a). Double staining with IBA-1 antibody, a marker of microglia/macrophage cells, revealed the localization of few microglia in the GCL and INL (Fig. 3b). The LPS injection induced a drastic increase of GFAP in the Müller glial cells, which was accompanied by an increase in microglial cells in the ganglion cell layer and inner nuclear layer (Fig. 3c–f). The activated microglia and Müller glia was substantially reduced after GTE treatments, which is more obvious for the high dose (550 mg/kg) (Fig. 3j–l) than low dose (275 mg/kg) treatments (Fig. 3g–i). Measurement of the fluorescence intensity of GFAP immunoreactivity in these retinal sections revealed a dose dependent reduction of GFAP staining in the retina (Fig. 3m), suggesting that GTE can suppress the activation of microglia and Müller glia in the retina after inflammation induction.

### GTE suppresses the expression of inflammatory cytokines in the retina and vitreous humor

**Figure 4:**
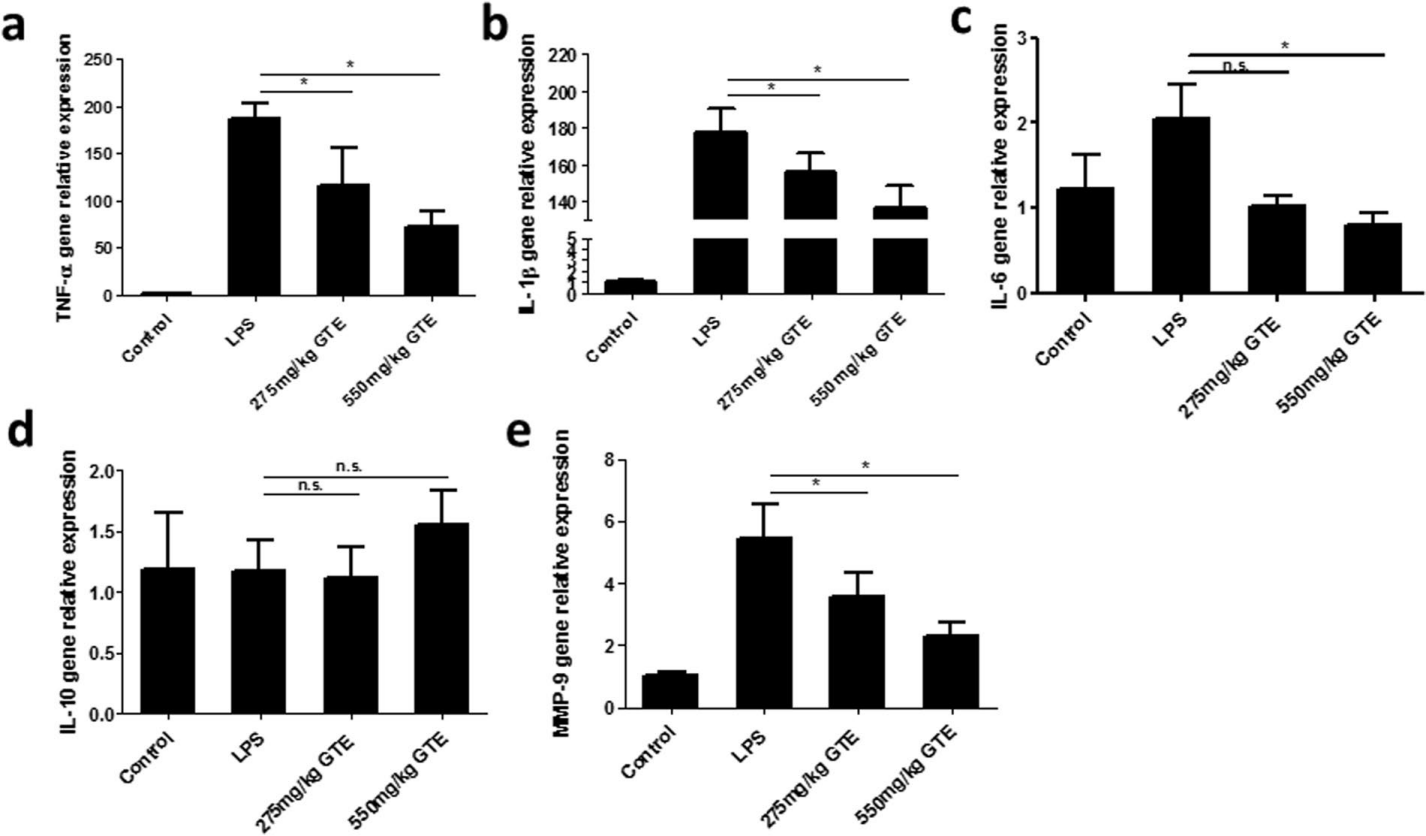
Green tea extract decreased the IL-1β, TNF-α, IL-6 and MMP9 gene expression in retina. (**a**–**c**) The retina inflammatory-related gene expression was detected by quantitative-PCR. The gene expression of IL-1β, TNF-α and IL-6 were elevated by LPS and inhibited by GTE intake. (**d**) However, the anti-inflammatory gene, IL-10 did not show any significant change in both LPS and GTE group. (**e**) Moreover, MMP9, a gene that regulates the migration ability was down regulated in GTE group compared to LPS group. *p < 0.05, n.s. no significantly difference, n = 7.

The anti-inflammatory effects of GTE were further investigated by the expression of inflammatory cytokines in the retina and vitreous humor. Quantitative PCR analysis showed that the pro-inflammatory factors TNF-α, IL-1β and IL-6 were all upregulated in the retina 48 hrs after LPS injection (Fig. 4a–c). Expression of these genes was suppressed significantly by GTE treatments in a dose dependent manner. On the other hand, IL-10, an anti-inflammatory cytokine, did not show any significant change after GTE treatments (Fig. 4d). Expression of MMP9, a matrix metalloproteinase important for migration and invasion, also showed a significant elevation in the retina after LPS insult (Fig. 4e). This elevation was reduced significantly after GTE treatments, suggesting that GTE may suppress migratory ability of inflammatory cells from the retina into the vitreous humor.

**Figure 5:**
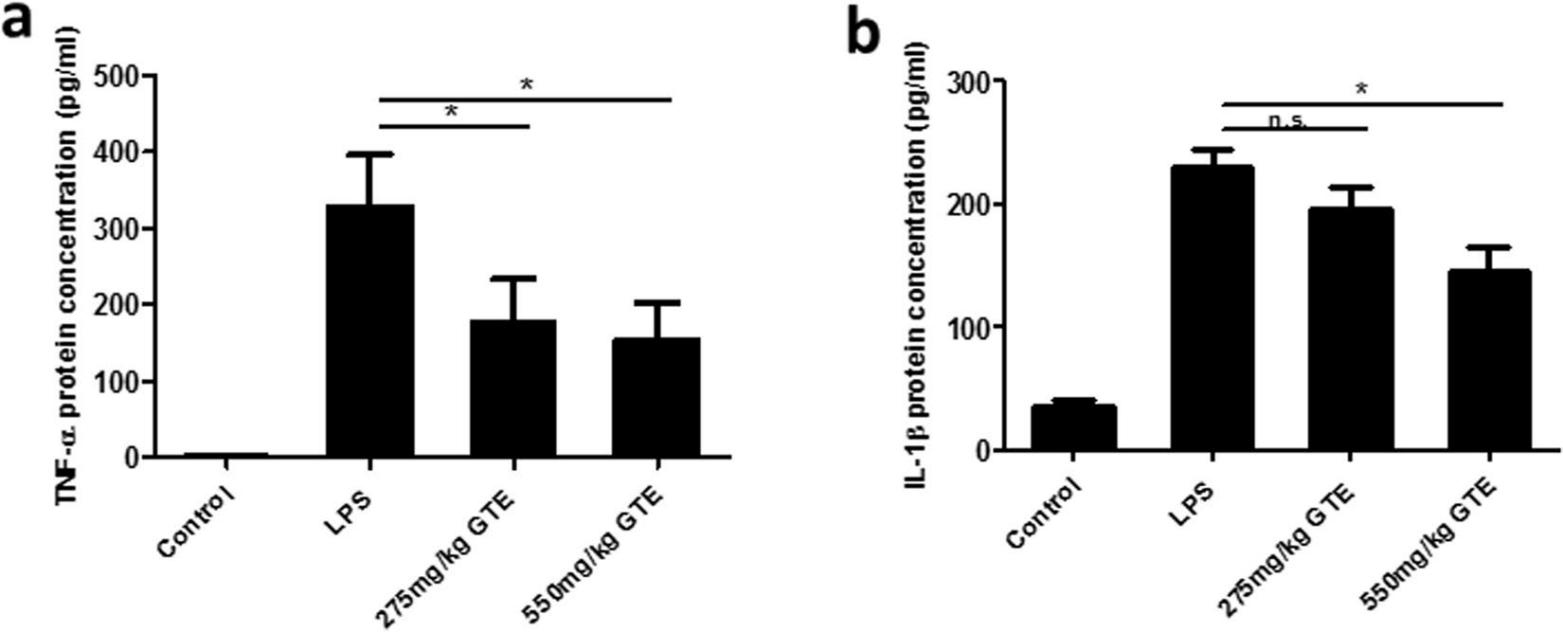
Green tea extract inhibited the IL-1β, TNF-α protein accumulation in vitreous humor. (**a**,**b**) The concentrations of pro-inflammatory factors TNF-α, IL-1β were increased significantly 48 hrs after LPS treatment. 550 mg/kg GTE produced a prominent reduction on the secretion of these two cytokines compared LPS group. However, the lower dosage group (275 mg/kg group) only had a lower TNF-α concentration but no statistical differences from the LPS group in the concentration of IL-1β. *p < 0.05, n = 7.

The expression and secretion of proinflammatory cytokines was also investigated in the vitreous humor using ELISA. The results showed that there was a surge in both TNF-α and IL-1β in vitreous humor 48 hrs after LPS treatment (Fig. 5a,b). GTE at the dosage of 550 mg/kg produced significant suppression to the increase in TNF-α and IL-1β and the effect was more prominent for the high dose than low dose of GTE.

### GTE attenuates the phosphorylation of STAT3 and NF-ĸB in LPS-induced rat retina

**Figure 6:**
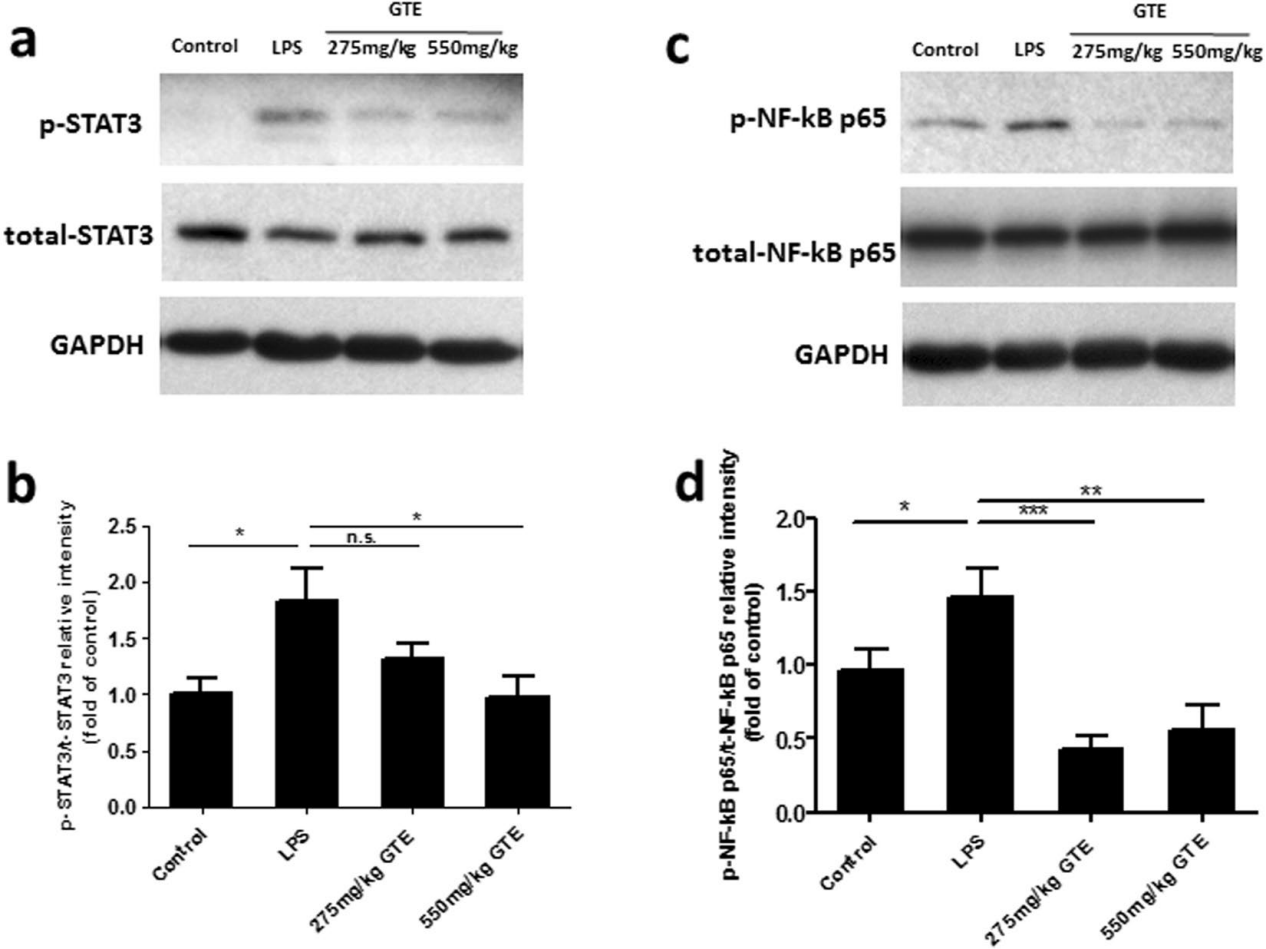
Green tea extract inhibited the phosphorylation of STAT3 and NF- κB p65 in LPS-induced rat retina. (**a**,**b**) Western blot results showed that the phosphorylation of STAT3 and NF- κB p65 (Ser 536) were substantially increased in LPS treated rats. GTE treatment at 275 mg/kg and 550 mg/kg suppressed their phosphorylation level. (**c**,**d**) Quantitative measurements of the bands showed that treatment of GTE remarkably attenuated the phosphorylation of STAT3 and NF- κB p65. **p < 0.01, ***p < 0.001, n = 7.

To determine the mechanisms of anti-inflammatory effects of GTE, the activation of STAT3 and NF-ĸB, the key molecules in promoting cytokine expression, was investigated. Western blot results showed that LPS caused a substantial increase in phosphorylation of STAT3 and NF-ĸB in the retina (Fig. 6a,b). Quantitative measurements of the bands showed that GTE treatments attenuated significantly the LPS-induced phosphorylation of STAT3 and NF-ĸB (Fig. 6c,d), supporting that these signaling molecules are mediating the anti-inflammatory actions of GTE.

### Localization of EGCG receptor 67LR in rat retina

**Figure 7:**
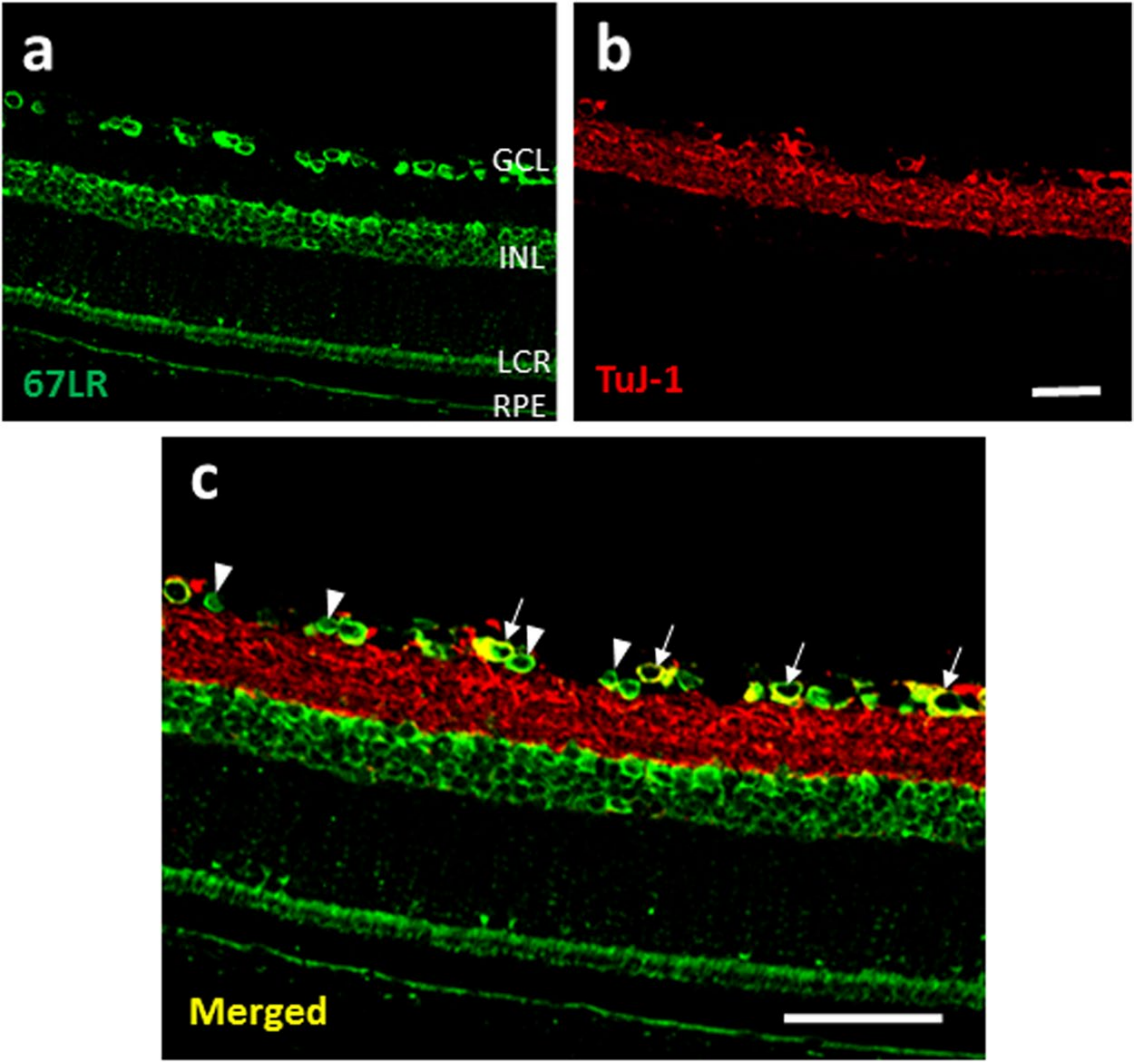
The receptor of EGCG, 67LR, was expressed in the retina. (**a**) 67LR staining was observed in GCL, INL, layer of cones and rods (LCR) and retinal pigment epithelium layer (RPE). (**b**,**c**) Some of the 67LR staining in GCL was co-localized with TUJ1 staining (white arrow), which recognizes the neuronal class III β-Tubulin in retinal ganglion cells and their dendrites. Meanwhile, the 67LR staining was also observed on some relatively smaller cells (arrowhead) in GCL, which are likely the glial cells. Scale bars: 40 µm, n = 4.

The non-integrin laminin receptor 67LR has been shown recently to be a cell surface receptor of EGCG, the major constituent of GTE. To determine whether this receptor is expressed in the retina, immunohistochemistry was used to determine the localization of 67LR protein in both normal and LPS induced adult rats. In the retina, 67LR was expressed in the βIII-tubulin-positive RGCs, and a number of presumptive astrocytes (Fig. 7a–c). Moderate staining was observed in the inner nuclear layer, layer of cones and rods, and the retinal pigment epithelium, suggesting a potential interaction of these cells to EGCG in the GTE. No obvious changes were found in LPS treated and normal retina (data not shown).

## Discussion

In the present study, we have demonstrated that oral administration of GTE produces a potent anti-inflammatory effect to the retina during LPS-induced inflammation. The major findings are: (i) GTE suppresses the infiltration of inflammatory cells and accumulation of protein in the vitreous humor that resulted from LPS insult; (ii) the activations of astrocytes, microglia and Müller glia during the inflammatory process are significantly attenuated by GTE treatments; (iii) these anti-inflammatory actions are associated with a reduction in synthesis and secretion of proinflammatory factors in the retina; (iv) GTE treatments ameliorate the phosphorylation of STAT3 and NF- κB in the retina; and (v) the EGCG receptor 67LR is mainly localized on the GCL and INL in the retina. These findings support a potent anti-inflammatory action of GTE in posterior segments of the eye during LPS induced acute inflammation.

One major finding in this study is that GTE can effectively suppress the proliferation of microglial cells 48 hours after LPS insult. Retinal microglia originate from hematopoietic progenitor cells and later differentiate into parenchymal microglia in the adult^22^. They are the intrinsic resident immune cells and rest in a dormant state in normal adult retinas^23^. Once activated under the pathological conditions, they immediately initiate the inflammatory response by generating cytokines like IL-1β and TNF-α^24^. Our results show that LPS activates the microglia and drives them to the RGC layer that is populated with astrocytes. The increase in microglia is likely produced by proliferation of resident microglia in the retina, and by recruitment of microglia from monocyte precursors in the blood vessels^25–28^, which causes the increase of cytokines in both retina and vitreous humor. Oral administration of GTE alleviates the recruitment and activation of microglia and so decreases the synthesis and secretion of proinflammatory factors IL-1β, IL-6 and TNF-α in the retina, but not of IL-10 that is known to be anti-inflammatory. These findings agree with previous results, which shows EGCG inhibits the release of NO and TNF-α from LPS-activated microglia^29^. Moreover, we have shown that expression of MMP-9, an enzyme essential for cell migration^30^, is reduced after GTE treatment, providing support to the function of GTE on suppressing the recruitment of microglia/monocytes into the retina.

Another important finding is that GTE treatments alleviate the activation of Müller glia in the retina after LPS treatment. In astrocytes, LPS induces changes in morphology in the activated astrocytes, which include cell hypertrophy and disruption of the glial endfeet that form part of the blood-ocular barrier^31^. The loss of blood-ocular barrier integrity probably results in influx of protein from the vessels into the vitreous humor, and recruitment of inflammatory cells from the systemic circulation into the retinal parenchyma and vitreous humor. The astrocytic reaction has been shown to regulate microglial activities during inflammation. It has been reported that the activated astrocytes exacerbate the inflammatory responses by enhancing NO production from the microglia during LPS insult^32,33^. Müller glia are another major glial cell type in the retina, which span the entire thickness of the retina and support the structure and stabilization of retinal neurons^34^. It has been shown that Müller glia help to amplify the inflammatory response and mobilize microglia in the retinal inflammation^35,36^. The reactive gliosis in the Müller glia is manifested by an upregulation of GFAP in a number of retinal diseases^37,38^. In the current study, we have shown that LPS induces an obvious increase in GFAP immunoreactivity in the Müller glia, which probably acts to stabilize newly formed terminal processes^38^. Treatments of GTE attenuate the increase in GFAP expression in Müller glial cells, which coincides with the gene expression and Western blotting results in previous studies^31,39^.

The anti-inflammatory actions of GTE are largely attributed to the anti-oxidative properties of the catechin constituents in the GTE. Our earlier reports have shown that retinal tissues are accessible to catechins through an oral gavage of GTE, which accumulate to sufficient amounts that protect the retina from oxidative insults^13,40^. Our recent study has shown that GTE at doses similar to the current study ameliorates the retinal degeneration in rats induced by sodium iodate^14^. These neuroprotective effects are associated with suppression of expression of superoxide dismutase and glutathione peroxidase, and a reduced level of 8-iso-prostaglandin F_2α_ production in the retina, indicating the potent anti-oxidative effect of GTE. We show further in the current study that the EGCG receptor, 67LR, is expressed prominently in different cell types in the retina, including RGC and retinal glia, suggesting that the GTE may exert its action through a receptor mediated mechanism, in addition to its anti-oxidative activities. Although the links between this receptor and downstream signaling pathways are not known, we have shown that GTE treatments produce a reduction in phosphorylation of STAT3 and NF-ĸB in the retina; both are known to mediate production of inflammatory cytokines. STAT3 is a transcription factor activated by tyrosine kinases. The phosphorylated STAT3 forms dimers and translocate into the nucleus. STAT3 cooperates with NF-ĸB to orchestrate the expression of a variety of inflammatory genes, such as IL-1β and TNF-α^41–43^. GTE or its component constitute EGCG has been reported to inhibit STAT3 signaling pathway in human pancreatic cancer and breast cancer cell lines^44,45^. In this study, the GTE constituent EGCG is likely mediating the anti-inflammatory actions through binding to 67LR in the retina that signals a suppressed phosphorylation of STAT3 and NF- κB that results in reduced production of inflammatory cytokines.

In summary, the results from our experiment support that LPS induces acute retinal inflammation in 48 hours by initiating inflammatory cell infiltration as well as activation of microglial cells and Müller glia. Oral administration of GTE alleviates activation of these cells and inhibits synthesis and secretion of inflammatory cytokines during inflammation. Hence, we conclude that GTE has a potent anti-inflammatory effect to the retina and can be considered as a potential therapeutic agent against acute retinal inflammation.

## Materials and Methods

### Green tea extract

The green tea extract Theaphenon E was kindly provided by Dr. Y. Hara. It contains a mixture of EGCG (epigallocatechin gallate, 70.53%), EGC (epigallate catechins, 4.61%), EC (epicatechin, 3.88%) and GC (gallocatechin, 0.64%), and other trace catechin derivatives^40^. It was prepared as a 550 mg/kg or 275 mg/kg suspension in 0.5 ml distilled water and was immediately fed intragastrically into the rat. The dosage of 550 mg/kg has been shown in our earlier study to be the optimal dose that produces potent anti-inflammatory effects against acute uveitis in the rat without causing obvious toxic effects to major organs^13^.

### Animal experiments

All animal experiments were performed following the guidelines of the Association for Research in Vision and Ophthalmology (ARVO) Statement on the Use of Animals in Ophthalmic and Vision Research. Ethics approval for the study was obtained from the Chinese University of Hong Kong Animal Ethics Committee. Fifty-two adult male Sprague-Dawley rats weighing at 230–250 g were obtained from the Laboratory Animal Service Center of the Chinese University of Hong Kong. Before the experimentation, animals were maintained with free access to food and water in an isolated room for at least 2 days to adapt to the environment. The rats were divided into four groups randomly. (i) Control: injected with saline into one footpad and fed with water 2, 8, 26, 32 hours after injection. (ii) LPS: injected with 1 mg/kg lipopolysaccharide (LPS; L2262, Sigma-Aldrich) into one footpad and fed with water at the same time points as stated above. (iii) 550 mg/ml GTE: injected with 1 mg/kg LPS and fed with 550 mg/ml GTE at the four time points. (iv) 275 mg/ml GTE: injected with 1 mg/kg LPS and fed with 275 mg/ml GTE at the four time points (n = 13 in each group). Forty-eight hours after LPS injection, rats were terminated by overdosing with ketamine-xylazine mixture. Six rats in each group were used for whole mount staining and H&E staining. The remaining seven rats were prepared for vitreous humor collection, retinal RNA and protein detection.

### Retinal Whole mount

Rats were anaesthetized by intraperitoneal injection of ketamine (35 mg/kg) and xylazine (5 mg/kg) and perfused with PBS. The retinas were dissected out in 4% paraformaldehyde (PFA) and mounted on a glass coverslip. After fixing for at least 1 hour at room temperature, the retinas were washed in PBS and incubated in PBS with 1% Triton X-100 at room temperature for 15 minutes. The retinas were blocked in 20% fetal calf serum for 1 hour, and then incubated with mouse anti-OX-42 polyclonal antibody at 1:100 dilution (Bio-Rad AbD Serotec; MCA275) and rabbit anti-GFAP polyclonal antibody at 1:1000 dilutions (Dako; Z0334) (in PBS-T with 0.9% NaCl and 0.5% BSA) overnight at 4 °C. On the following day, the retinas were washed with PBS-T/NaCl/BSA and incubated in goat anti-mouse IgG–FITC and goat anti-rabbit IgG–AF568 antibodies (Alexa Fluor Company) in PBS-T/NaCl/BSA at 1:200 for 2 hours at room temperature under dark condition. Finally, the retinal tissues were washed with PBS and mounted onto coverslips with glycerol. Imaging was performed using a confocal microscope (FV300, Olympus Co, Japan). Captured images were used for cell counting with Nikon NIS-Elements BR Analysis software. Twelve areas were selected from each retina and the mean numbers were compared for statistical analysis.

### Paraffin sectioning and immunostaining

Rats were perfused with 200 ml PBS and 200 ml 4% PFA. The eyeballs were fixed in PFA for 24 hours before processing with paraffin embedding. The blocks were cut into 5 µm sections, and transverse sections of the retina cutting through the optic disk were collected. Before performing the immunostaining, slides were processed with antigen retrieval in Tris-EDTA Buffer (1 mM EDTA Solution, 10 mM Tris Base, 0.05% Tween 20, pH = 9.0) at 110 °C for 10 min. Slides were cooled down and washed with distilled water before incubation with 10% normal goat serum in PBS with 0.01% Triton X-100. Primary and secondary antibodies were diluted in 1% normal goat serum and tissues were incubated for 2 hrs and 1 hr, respectively, at room temperature. Fluorescence signals were visualized by confocal microscopy (FV300, Olympus Co, Japan) with 40X lens.

### Western blotting

The protocol for Western blotting was described in our previous experiments^46^. Only minor modifications were made. Briefly, the retina was dissected out from the eyeball and lysed in equal volume fresh 1× RIPA buffer (Millipore, 20–188) and proteinase inhibitor. After incubation for 30 minutes on ice, the tubes were centrifuged at 12000 rpm for 30 minutes at 4 °C to obtain the protein from the supernatant fraction. After measuring the protein concentration, 3 × loading buffer was added into the supernatant and heated for 10 minutes at 95 °C. 25 ug protein was loaded and resolved in 12% SDS gel and transferred onto a PVDF membrane (Advansta, L-08008-001). Membrane was blocked with 5% non-fat milk (Bio-Rad, 170–6404) in TBST solution and then incubated with primary antibodies (supplementary Table 1) overnight at 4 °C. On the second day, the membrane was washed with TBST for 3 times and then incubated with HRP-conjugated secondary antibody at 1:5000 for 1 hour at room temperature. The specific bands were visualized with chemiluminescence reagent and the exposure to the X-ray films. Band intensities of p-STAT3 was normalized to total-STAT3 with Gene Tools from Syngene software.

### Gene expression analysis

RNA in the retinas was collected following the protocol of the RNA extraction kit (Favorgen Biotech Corp, FATRK001) and then underwent reverse transcription into cDNA with a SuperScript III reverse transcriptase (Invitrogen, USA). Relative gene expression of mRNA was determined by ABI Quantstudio Flex Real Time PCR machine. The primers we used were listed in (Supplementary Table 2).

### Cell counting and protein assay in vitreous humor

We used a 1 ml insulin syringe (30-gauge needle) to collect the vitreous humor. 1 µl of vitreous humor was diluted at 1:10 with PBS and suspended in an equal volume of Trypan-blue solution to do the cell counting with the hemacytometer. The rest volume was centrifuged at 1500 rpm for 15 minutes at 4 °C. Cell-free supernatant was collected to detect the total protein concentration with the commercial product from Bio-Rad (Bio-Rad, Hercules, CA, USA).

### Determination of inflammatory cytokines in vitreous humor

ELISA experiments were performed to detect the specific protein concentration accumulated in the vitreous humor. Rat IL-1β and TNF-α ELISA kits were purchased from R&D Systems (RLB00, RTA00). 5 µl vitreous humor of each rat was diluted into 45 µl dilution buffer provided by the kit according to datasheet. All the other steps of the assays were performed according to the manufacturer’s instructions.

### Statistical analysis

All data was analyzed by nonparametric Kruskal-Wallis test followed by Dunn-Bonferroni post-hoc comparison. All the data was presented as mean ± SEM. The level of statistical significance was *p* < 0.05. All statistical analyses were performed using SPSS statistical software package (version 20.0).

## Electronic supplementary material

Supplementary information
