# Supplementary material for: Green tea extract attenuates LPS-induced retinal inflammation in rats
Source: Sci Rep. 2018 Jan 11;8:429. doi: 10.1038/s41598-017-18888-5 (PMC5765135; doi:10.1038/s41598-017-18888-5)
Supplement: Supplementary file 1 — Supplementary information [file 41598_2017_18888_MOESM1_ESM.doc]

**Green tea extract attenuates LPS-induced retinal inflammation in rats**

Jia Lin Ren^1^, Qiu Xiao Yu^1^, Wei Cheng Liang^1^, Pui Ying Leung^1^, Tsz Kin Ng^2^, Wai Kit Chu ^2^, Chi Pui Pang^2^, Sun On Chan^1^

Affiliations: 1. School of Biomedical Sciences, Faculty of Medicine, The Chinese University of Hong Kong. Shatin, N.T., Hong Kong SAR, China.

2. Department of Ophthalmology and Visual Sciences, Faculty of Medicine, The Chinese University of Hong Kong, Kowloon, Hong Kong SAR, China.

**Correspondence:**

Sun-On Chan, D.Phil., School of Biomedical Sciences, Faculty of Medicine, The Chinese University of Hong Kong, Shatin, N.T., Hong Kong SAR, China.

Phone: +852-39436898

Fax: +852-2603 5123

Email: [sunonchan@cuhk.edu.hk](mailto:sunonchan@cuhk.edu.hk)

**Supplementary table 1: Antibodies for protein expression.**

| **Protein** | **Company** | **Catalog number** | **Source** | **Dilution factor** |
| --- | --- | --- | --- | --- |
| GFAP | DAKO | Z0334 | rabbit | 1:1000 |
| OX-42 | Serotec | MCA275 | mouse | 1:100 |
| IBA-1 | Millipore | MABN92 | mouse | 1:100 |
| p-STAT3 | Cell Signaling | 9145S | rabbit | 1:1000 |
| Total STAT3 | BD Transduction Laboratories | S21320 | mouse | 1:1000 |
| p-NF-kB p65 | Immunoway | YP0191 | rabbit | 1:1000 |
| Total NF-kB | Santa Cruz | Sc-372 | rabbit | 1:500 |
| GAPDH | Ambion | MAB2916 | mouse | 1:100000 |

**Supplementary table 2: Primers for gene expression analysis (for rat).**

| **Gene** | |  | **Primer Sequence (5' > 3')** |  | |
| --- | --- | --- | --- | --- | --- |
| *IL-1β* | *F:* | | CTTTCATCACACAGGACAGG | |  |
|  | *R:* | | GTGATGTTCCCATTAGACAGC | |  |
| *MMP9* | *F:* | | CTGCCTGCACCACTAAAGG | |  |
|  | *R:* | | GAAGACGAAGGGGAAGACG | |  |
| *TNF-α* | *F:* | | CACGCTCTTCTGTCTACTGA | |  |
|  | *R:* | | GGACTCCGTGATGTCTAAGT | |  |
| *IL-6* | *F:* | | GACTGATGTTGTTGACAGCCACTGC | |  |
|  | *R:* | | TAGCCACTCCTTCTGTGACTCTAACT | |  |
| *IL-10* | *F:* | | AAAGCAAGGCAGTGGAGCA | |  |
|  | *R:* | | TCAAACTCATTCATGGCCTTGT | |  |
| *β-actin* | *F:* | | CCCTGTGCTGCTCACCGA | |  |
|  | *R:* | | ACAGTGTGGGTGACCCCGTC | |  |
